# Symptom profiles and accuracy of clinical case definitions for COVID-19 in a community cohort: results from the Virus Watch study

**DOI:** 10.12688/wellcomeopenres.17479.1

**Published:** 2022-03-10

**Authors:** Ellen Fragaszy, Madhumita Shrotri, Cyril Geismar, Anna Aryee, Sarah Beale, Isobel Braithwaite, Thomas Byrne, Max T. Eyre, Wing Lam Erica Fong, Jo Gibbs, Pia Hardelid, Jana Kovar, Vasileios Lampos, Eleni Nastouli, Annalan M.D. Navaratnam, Vincent Nguyen, Parth Patel, Robert W. Aldridge, Andrew Hayward, Virus Watch Collaborative

**Affiliations:** 1 Centre for Public Health Data Science, Institute of Health Informatics, University College London, London, UK; 2 Department of Infectious Disease Epidemiology, London School of Hygiene and Tropical Medicine, London, UK; 3 Institute of Epidemiology and Health Care, University College London, London, UK; 4 Centre of Health Informatics, Computing and Statistics, Lancaster University, Lancaster, UK; 5 Liverpool School of Tropical Medicine, Liverpool, UK; 6 Institute for Global Health, University College London, London, UK; 7 Population, Policy and Practice Research and Teaching Department, University College London, London, UK; 8 Department of Computer Science, University College London, London, UK; 9 Francis Crick Institute, London, UK; 10 Department of Population, Policy and Practice, UCL Great Ormond Street Institute of Child Health, University College London, London, UK

**Keywords:** COVID-19, symptoms, clinical case definitions

## Abstract

**Background: **Understanding symptomatology and accuracy of clinical case definitions for community COVID-19 cases is important for Test, Trace and Isolate (TTI) and future targeting of early antiviral treatment.

**Methods:** Community cohort participants prospectively recorded daily symptoms and swab results (mainly undertaken through the UK TTI system).  We compared symptom frequency, severity, timing, and duration in test positive and negative illnesses.  We compared the test performance of the current UK TTI case definition (cough, high temperature, or loss of or altered sense of smell or taste) with a wider definition adding muscle aches, chills, headache, or loss of appetite.

**Results:** Among 9706 swabbed illnesses, including 973 SARS-CoV-2 positives, symptoms were more common, severe and longer lasting in swab positive than negative illnesses.  Cough, headache, fatigue, and muscle aches were the most common symptoms in positive illnesses but also common in negative illnesses. Conversely, high temperature, loss or altered sense of smell or taste and loss of appetite were less frequent in positive illnesses, but comparatively even less frequent in negative illnesses.  The current UK definition had 81% sensitivity and 47% specificity versus 93% and 27% respectively for the broader definition. 1.7-fold more illnesses met the broader case definition than the current definition.

**Conclusions: **Symptoms alone cannot reliably distinguish COVID-19 from other respiratory illnesses. Adding additional symptoms to case definitions could identify more infections, but with a large increase in the number needing testing and the number of unwell individuals and contacts self-isolating whilst awaiting results.

## Introduction

The natural history of severe acute respiratory syndrome virus-2 (SARS-CoV-2) infection can range from asymptomatic infection in around 25% of cases ^1^ to severe or fatal Coronavirus disease-2019 (COVID-19) at a rate that is highly age dependent ^2^. Understanding the natural history of symptomatic COVID-19 in the community is critical to the control of infection because it informs decisions about who should seek and be given access to testing, which symptoms should prompt self-isolation, and whether those in contact with symptomatic people should self-isolate. Understanding the normal course of symptoms is also potentially helpful to patients and clinicians assessing whether care needs to be escalated due to unexpectedly severe or prolonged symptoms. In future, symptom profiles may also trigger early use of antivirals to prevent deterioration and potentially minimise transmission ^3^. Finally, understanding symptom profiles is important to inform syndromic surveillance. 

A wide range of clinical case definitions for COVID-19 are available utilising different combinations of symptoms to alert individuals to the need for testing, isolation and contact tracing. For example, the World Health Organization (WHO) include the following symptoms in the clinical case definition of a suspected case: acute onset of fever AND cough; OR acute onset of ANY THREE OR MORE of the following signs or symptoms: fever, cough, general weakness/fatigue, headache, myalgia, sore throat, coryza, dyspnoea, anorexia/nausea/vomiting, diarrhoea, altered mental status ^4^. The European Centre for Disease Control and Prevention (ECDC) defines a possible case based on at least one of cough, fever, shortness of breath or sudden loss of sudden onset of anosmia, ageusia or dysgeusia ^4^, and US Centers for Disease Control and Prevention (CDC) use the following clinical criteria in the absence of a more likely diagnosis: at least two of fever (measured or subjective), chills, rigors, myalgia, headache, sore throat, nausea or vomiting, diarrhoea, fatigue, congestion or runny nose OR any one of cough, shortness of breath, difficulty breathing, new olfactory disorder, new taste disorder OR severe respiratory illness with at least one of the following: clinical or radiographic evidence of pneumonia, acute respiratory distress syndrome ^5^.

In the UK, the Test Trace and Isolate community testing programme (TTI) currently asks individuals to seek testing if they have any of the following symptoms: a new continuous cough, a high temperature, loss of or altered sense of smell or taste ^6^.

Although it is clear that addition of further symptoms to case definitions could increase sensitivity (i.e. the proportion of all illnesses testing positive who met the definition), this is likely to be at the cost of reduced specificity (i.e. the proportion of all those illnesses testing negative who did not meet the case definition) and increasing numbers of people who require testing, isolation and contact tracing ^7^.

For example, recent analysis of data from the REACT community survey suggests that adding in loss of appetite, chills, headache or muscle aches could increase the proportion of cases identified from 53% to 75% but at the cost of a 2.7-fold increase in required testing capacity ^8^. Altering clinical case definitions will have different implications at varying levels of prevalence, since positive and negative predictive values of tests depend upon disease prevalence as well as test sensitivity and specificity. The timing within the course of an illness at which people infected with SARS-CoV-2 meet the case definition is important since earlier isolation of cases and contacts could reduce transmission, particularly as viral loads peaks early in the course of illness ^9^.

Prospective community studies where participants record symptoms in near-real time are needed to accurately measure COVID-19 symptom profiles with minimal recall bias. Here we describe prospectively recorded symptom profiles (frequency, severity and duration) of illnesses that tested positive for COVID-19 and illnesses that tested negative within a large community cohort study ( Virus Watch). We compare the test characteristics (sensitivity, specificity, positive and negative predictive values, timing of meeting the case definition, number needed to test to identify one case) for the current UK definition and a wider definition proposed following analysis of the REACT study, which adds headache or chills or loss of appetite or muscle aches to the existing UK definition. We explore how symptom profiles and case definition performance vary by age and stage of the pandemic.

## Methods

### Study design and data collection

The Virus Watch study is a prospective, community cohort study which has been following entire households in England and Wales since 22 June 2020, over the COVID-19 pandemic. As of 20 June 2021, 24328 households and 50,816 people across England and Wales have joined the study. The full study protocol is published elsewhere ^10^.

In brief, participating households completed a baseline survey at enrolment into the study, followed by prospective, detailed daily symptom diaries recording the presence and severity of any symptoms of acute respiratory and gastrointestinal infections during any episodes of illness occurring during follow-up. At the end of each week of follow-up, households were emailed a survey link to report any symptoms from the preceding week as well as results of any SARS-CoV-2 swab tests conducted, including testing through TTI and routine asymptomatic screening, and including both polymerase chain reaction (PCR) and lateral flow device testing. Within the main cohort there was a nested sub-cohort of 10,766 participants who additionally provided study-specific swab specimens following predetermined symptom triggers ( *Extended data:* Additional methods ^11^) tested for SARS-CoV-2 by real-time reverse transcriptase PCR (RT-PCR) from the end of December 2020 onwards. 

Symptom data gathered through the weekly survey were grouped into illness episodes and matched to swab results (see *Extended data:* Additional methods). The start date of an illness episode was defined as the first day any symptoms were reported, and the end date was the final day of reported symptoms. A 7-day washout period where no symptoms were reported was used to define the end of one illness episode and the start of a new illness episode. The data presented in this analysis includes illnesses which began between 22 June 2020 (start of the study) through to 20 June 2021. Within illness episodes, we investigated a wide range of individual symptoms (see *Extended data:* Additional methods) and the following symptom groupings: **UK Case definition** – one or more of the following: cough, measured fever (≥37.8C) or feeling feverish, or loss of, or altered, sense of smell or taste. **Broader case definition** - one or more of the following: cough, measured fever (≥37.8C) or feeling feverish, loss of, or altered, sense of smell or taste, headache, muscle aches, loss of appetite or chills.

### Analysis

We present simple descriptive analyses of the frequency, severity and duration of symptoms in SARS-CoV-2 test positive and test negative illnesses. We calculated sensitivity and specificity for individual symptoms and, for the two case definitions, we also calculated the positive predictive value (PPV), negative predictive value (NPV) number of people meeting the definition within the cohort and the numbers needed to test to identify one positive case (NNT) ( *Extended data:* Figure S1 ^11^). 

We calculated the proportion of illnesses with SARS-CoV-2 test results and how this varied by UK case definition status and over time. We also calculated the ratio of the proportion of illnesses getting tested that met the UK case definition to those that did not meet the UK case definition. For both of these estimates we excluded illnesses beginning before 28 September 2020 as prior to this, our weekly surveys did not ask participants to report test results in weeks where there were no reported symptoms ( *Extended data:* Additional methods). This could have resulted in omission of some test results, particularly if there was a lag between swabbing and receipt of results. We also excluded illnesses beginning in the final week of data collection (week commencing 26 April 2021) as there may have been insufficient time to receive and report swab results. The analysis was conducted using the statistical software STATA MP 16 (StataCorp, 2019). 

### Ethics

The Virus Watch study has been approved by the Hampstead NHS Health Research Authority Ethics Committee (Ethics approval number - 20/HRA/2320). Written informed consent for the collection and use of data was obtained from participants.

## Results

Overall, there were 31,962 illnesses (with at least one of the symptoms, as defined in *Extended data:* Table S1 ^11^) reported in the cohort between 22 June 2020 and 20 June 2021. 9706 (30.4%) illnesses were swabbed and reported a corresponding result and of these 973 (10.0%) tested positive for SARS-CoV-2; 465 of these swabs were conducted as part of the study, including 23 positives. The percentage of swabbed illnesses testing positive were highest in young adults aged 16-24 years and lowest in children aged 0-15 years; highest in London and lowest in the South East and South West regions; and peaked in December 2020 ( Table 1). Those with symptoms meeting the UK case definition for swabbing by TTI were more likely to be swabbed than those with other symptoms ( *Extended data:* Table S2 ^11^).

**Table 1.** Characteristics of illnesses by demographics, VOC hotspots and swab outcome.

	All illnesses		Swabbed illnesses	Swab+ illnesses	
	N	% of all illnesses (column %)	N	N	% of swabbed illnesses (row %)
Overall	31962	100%	9706	973	10%
By Age Group **					
0–15	4618	15%	1397	83	6%
16–24	943	3%	344	59	17%
25–44	7281	23%	2505	278	11%
45–64	11310	36%	3687	384	10%
65+	7538	24%	1702	167	10%
By Sex ***					
Male	10822	36%	3076	369	12%
Female	19531	64%	6171	536	9%
By Region ****					
East Midlands	2851	9%	953	96	10%
East of England	5369	17%	1586	150	9%
London	4511	15%	1516	195	13%
North East	1622	5%	422	44	10%
North West	3508	11%	1136	138	12%
South East	5927	19%	1812	124	7%
South West	2475	8%	616	52	8%
Wales	786	3%	180	20	11%
West Midlands	1952	6%	604	55	9%
Yorkshire and The Humber	1841	6%	565	60	11%
By Month *****					
Jun-Aug 2020	1869	6%	203	5	2%
Sep-20	5646	18%	694	52	7%
Oct-20	3895	12%	876	105	12%
Nov-20	3407	11%	967	120	12%
Dec-20	4160	13%	1647	355	22%
Jan-21	2860	9%	1236	215	17%
Feb-21	2490	8%	804	53	7%
Mar-21	2724	9%	971	29	3%
Apr-21	1975	6%	863	9	1%
May-21	2178	7%	1080	14	1%
Jun-21	758	2%	365	16	4%

* Strain hotspot not classifiable or between classifiations for 7022 illnesses ** Age missing for 272 illnesses *** Sex missing for 1609 illnesses **** Region missing for 1120 illnesses ***** Month missing for 0 illnesses Note 1: The last 3 columns are currently limited to the first swab positive illness if a person has multiple illnesses that merged with a positive swab. We are currently dropping 68 illnesses that were 2nd, 3rd of 4th swab+ illnesses for a person from these coloumns. These 68 illnesses are still included in the other coloumns though. Although we could investigate this - we don't currently know if these were different swabs testing positive or the same positive swab merging with two illnesses close in time. Note 2: The swabbed illnesses column is really just swabbed illnesses which we have a result for. We know of some illnesses which were swabbed which we never got a result for and those are not included in this coloumn.

When excluding illnesses without full opportunity to report swab results, the proportion of illnesses reporting swab results steadily increases from September 2020 to January 2021, temporarily drops in February and increases again through May 2021 and begins to dip again in June 2021 ( *Extended data:* Figure S2 ^11^). Illnesses meeting the case definition were more likely to be swabbed than illnesses not meeting the case definition, although the differences in swabbing behaviour of these two groups changed over time ( *Extended data:* Figures S2 & S3). The ratio of the proportion of illnesses reporting a test result among those meeting the case definition to those not meeting the case definition was highest in September 2020, when those meeting the case definition were 4.4 times more likely to report a test result. This ratio steadily declined over time to its lowest point in May 2021 when illnesses meeting the case definition were only 1.4 more likely to report a test result compared to illnesses not meeting the case definition. 

**Figure 1.**
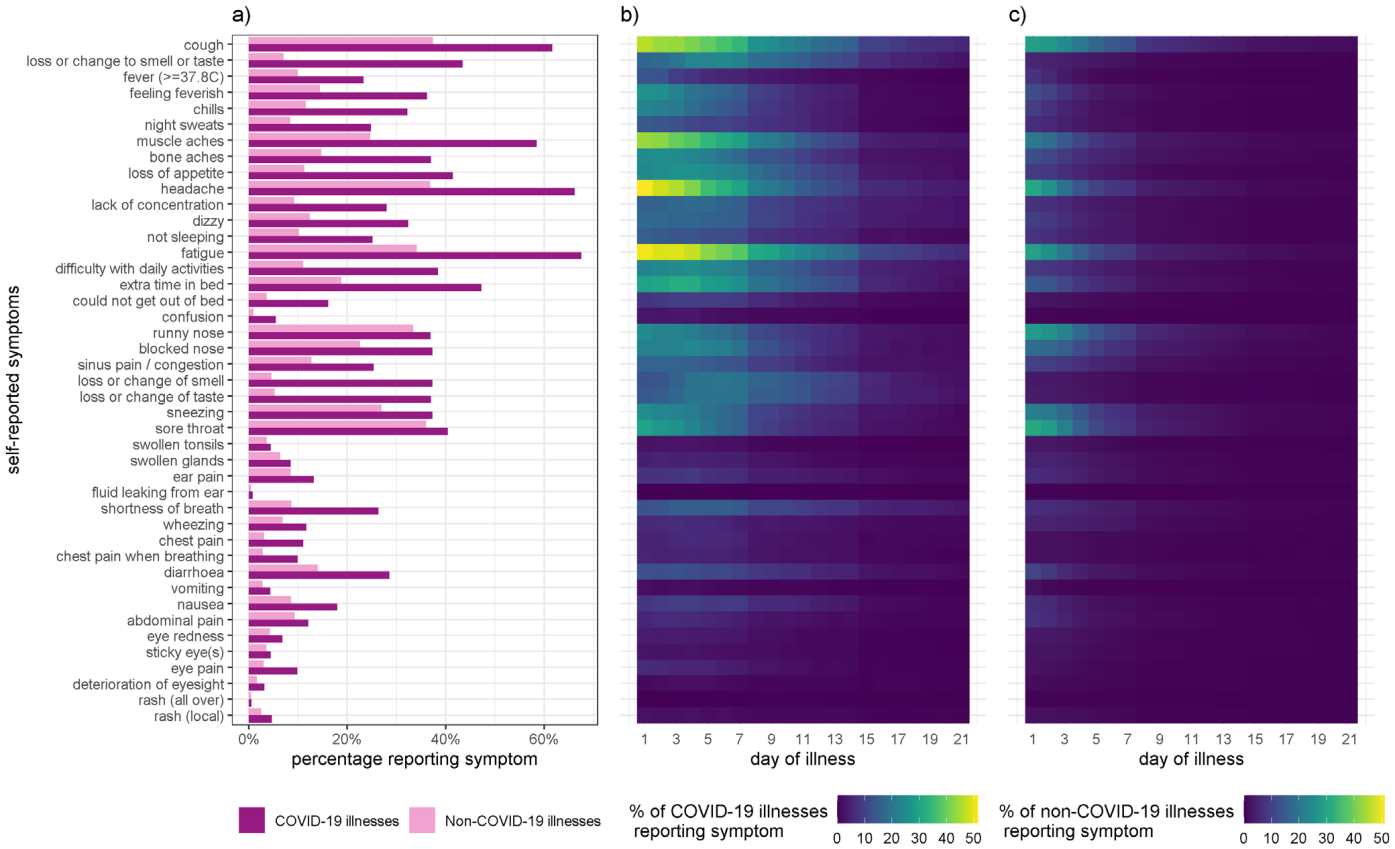
COVID-19 symptoms. **a**, Self-reported symptoms by swab-positive and swab-negative illnesses. **b**- **c**, Proportion of COVID-19 illnesses ( **b**) and non-COVID-19 illnesses ( **c**) experiencing symptoms on a given day of illness within the first three week of illness. Day 1 represents the onset of symptoms.

Figure 1a shows the proportion of swabbed illnesses that reported each symptom according to whether they tested positive or negative for SARS-CoV-2. Almost all of the wide range of reported symptoms were more common in illnesses that tested positive than illnesses that tested negative. Amongst SARS-CoV-2-positive illnesses, the most commonly reported symptoms, in decreasing order of frequency, were: fatigue, headache, cough, muscle ache, needing to spend extra time in bed, loss of, or altered, sense of smell or taste, loss of appetite, sore throat, and difficulties in undertaking daily activities. The percentage of illnesses with each symptom by day of illness is shown in Figures 1b and c, which illustrates both the higher frequency and longer duration of key symptoms in test positive and test negative cases.

Table S2 ( *Extended data*) shows the sensitivity and specificity of each symptom and the mean and median day of illness on which they are first reported among all swabbed illnesses with a reported result. Among all illnesses reported (including non-swabbed and SARS-CoV-2 negative illnesses), most symptoms arise early in the course of illness (mean onset typically on day 2) although loss or change to sense of smell or taste and the two chest pain symptoms appear slightly later (mean onset late in day 2 to middle of day 3 respectively) during the course of illness. It can be seen that although cough and some constitutional symptoms such as headache, fatigue and muscle aches are fairly common among COVID-19 illnesses (i.e., they have a moderate sensitivity), they are also a common feature of non-COVID-19 illnesses and thus do not have a high specificity. In contrast the loss or change to sense of smell or taste is slightly less common among COVID-19 illnesses but comparatively much less common among non-COVID-19 illnesses, leading to a lower sensitivity but higher specificity.

**Figure 2.**
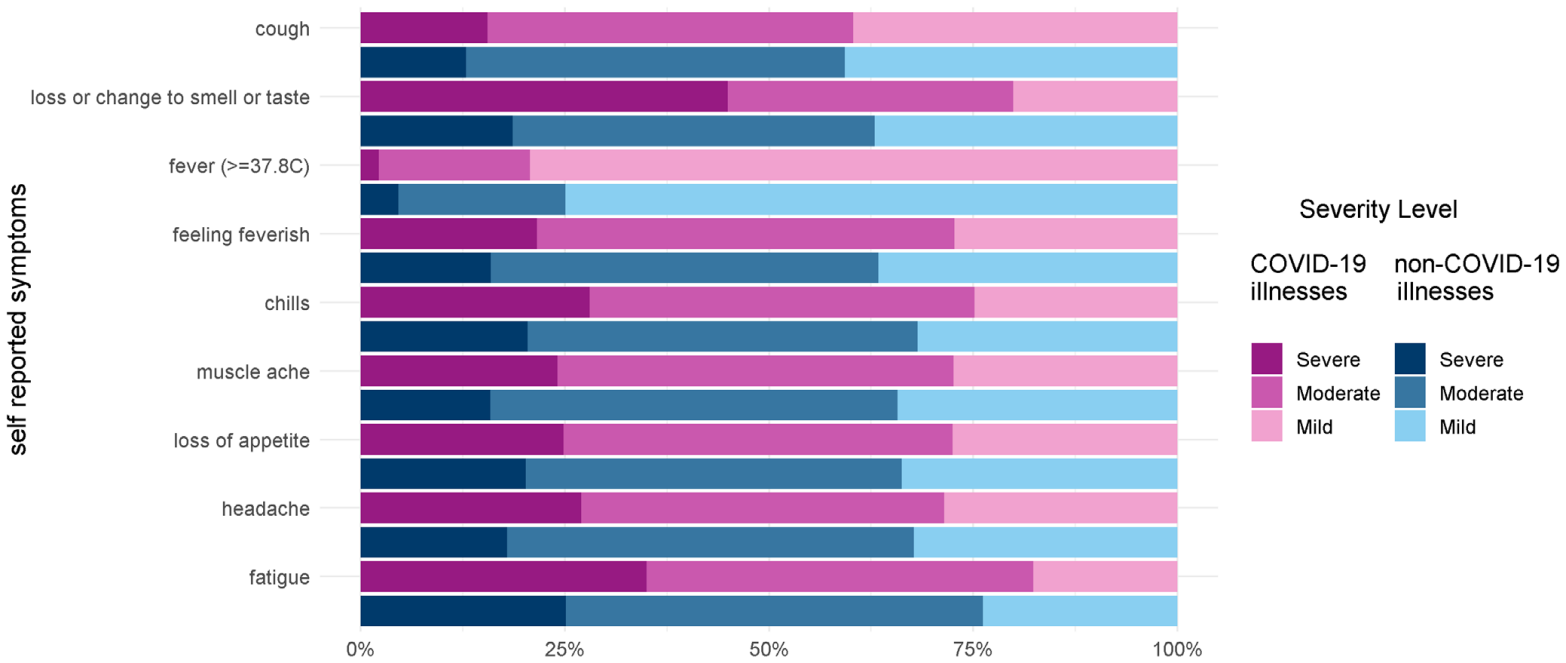
Severity of symptoms among COVID-19 illnesses and non-COVID-19 illnesses reporting each symptom.

**Figure 3.**
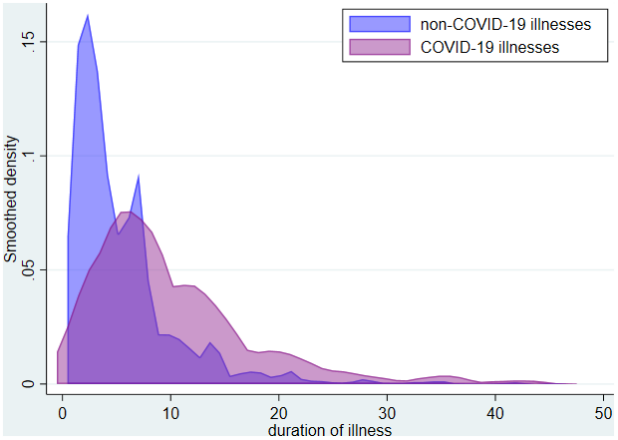
Distribution of illness duration* by COVID-19 status. *limited to illnesses with duration <50 days.

Figure 2 and Table S3 ( *Extended data* ^11^) show the maximum reported severity for a range of key symptoms in test positive and test negative illnesses. When symptoms do occur, they are more likely to be severe in COVID-19 illnesses than in non-COVID-19 illnesses. Figure 3 shows the distribution of the duration of illnesses in test positive and test negative illnesses. It can be seen that the duration of illness is longer in COVID-19 illnesses than in other illnesses. Table S4 ( *Extended data* ^11^) shows how illness duration varies by age and sex in COVID-19 and non-COVID-19 illnesses and Figure S4 ( *Extended data* ^11^) shows the distribution of duration of illnesses among COVID-19 illnesses by age group. Illnesses tend to be of longer duration in older individuals.

Table 2 shows the mean and median day of meeting the current and broader case definition, sensitivity, specificity, PPV, NPV, and NNT to identify a case. The numbers meeting the case definition and how many folds higher this is for the broader case definition are shown (multiplication factor column). These results are stratified by age group and calendar time. 

**Table 2.** Speed of identifying cases, proportion of all community cases requiring testing and test characteritics for the current and proposed UK Test and Trace case definitions.

Case Definition	Strata		Onset Mean	Onset Median	Sensitivity	Specificity	PPV	NPV	NNT	N illnesses eligible for testing	Multiplication factor
**Current** ** Case ** **Definition**	**Overall**	Overall	1.8	100%	**81%** (79%, 84%)	**47%** (46%, 48%)	**15%** (14%, 16%)	**96%** (95%, 96%)	**7**	**11340**	**1**
	**by age ** **group**	0–15	1.7	100%	**63%** (51%, 73%)	**29%** (26%, 31%)	**5%** (4%, 7%)	**92%** (89%, 95%)	**19**	**1858**	**1**
		16–44	1.9	100%	**84%** (80%, 88%)	**48%** (46%, 50%)	**18%** (16%, 20%)	**96%** (94%, 97%)	**6**	**3000**	**1**
		45–64	1.8	100%	**84%** (80%, 87%)	**53%** (51%, 54%)	**17%** (15%, 19%)	**96%** (96%, 97%)	**6**	**3875**	**1**
		65+	1.8	100%	**80%** (73%, 85%)	**50%** (48%, 53%)	**15%** (13%, 17%)	**96%** (94%, 97%)	**7**	**2489**	**1**
	**by** ** time** ** period**	Jun-Sep	2.4	100%	**81%** (68%, 90%)	**31%** (28%, 34%)	**7%** (5%, 10%)	**96%** (93%, 98%)	**14**	**2432**	**1**
		Oct-Nov	1.8	100%	**85%** (80%, 89%)	**41%** (39%, 44%)	**17%** (15%, 19%)	**95%** (93%, 97%)	**6**	**2417**	**1**
		Dec-Jan	1.7	100%	**82%** (79%, 85%)	**46%** (44%, 48%)	**27%** (25%, 30%)	**91%** (90%, 93%)	**4**	**2755**	**1**
		Feb-Apr	1.6	100%	**66%** (55%, 76%)	**56%** (54%, 57%)	**5%** (4%, 6%)	**98%** (97%, 99%)	**20**	**2477**	**1**
		May-Jun	1.5	100%	**83%** (65%, 94%)	**49%** (46%, 52%)	**3%** (2%, 5%)	**99%** (98%, 100%)	**30**	**1259**	**1**
**Proposed ** **Case ** **Definition**	**Overall**	Overall	1.5	100%	**93%** (91%, 95%)	**27%** (26%, 28%)	**12%** (12%, 13%)	**97%** (97%, 98%)	**8**	**18970**	**1.67**
	**by age ** **group**	0–15	1.6	100%	**81%** (71%, 89%)	**22%** (20%, 25%)	**6%** (5%, 8%)	**95%** (92%, 97%)	**16**	**2275**	**1.22**
		16–44	1.5	100%	**93%** (90%, 96%)	**27%** (25%, 29%)	**15%** (13%, 16%)	**97%** (95%, 98%)	**7**	**5046**	**1.68**
		45–64	1.4	100%	**95%** (93%, 97%)	**27%** (25%, 28%)	**13%** (12%, 14%)	**98%** (97%, 99%)	**8**	**7092**	**1.83**
		65+	1.5	100%	**94%** (89%, 97%)	**29%** (27%, 32%)	**13%** (11%, 15%)	**98%** (96%, 99%)	**8**	**4387**	**1.76**
	**by** ** time ** **period**	Jun–Sep	1.7	100%	**89%** (78%, 96%)	**18%** (15%, 20%)	**7%** (5%, 9%)	**96%** (92%, 99%)	**15**	**4065**	**1.67**
		Oct–Nov	1.5	100%	**95%** (91%, 97%)	**20%** (19%, 23%)	**14%** (12%, 16%)	**97%** (94%, 98%)	**7**	**4122**	**1.71**
		Dec–Jan	1.4	100%	**94%** (92%, 96%)	**27%** (25%, 29%)	**24%** (22%, 26%)	**95%** (93%, 96%)	**4**	**4235**	**1.54**
		Feb–Apr	1.3	100%	**89%** (81%, 95%)	**31%** (29%, 33%)	**4%** (4%, 5%)	**99%** (98%, 99%)	**23**	**4663**	**1.88**
		May–Jun	1.3	100%	**87%** (69%, 96%)	**31%** (29%, 34%)	**3%** (2%, 4%)	**99%** (98%, 100%)	**38**	**1885**	**1.5**

Sensitivity of the current UK case definition was 81% (95% CI 79-84%) compared to 93% (95% CI 91-95%) for the broader case definition. Specificity was 47% (95% CI 46-48%) for the current case definition and 27% (95% CI 26-28%) for the broader case definition. Sensitivity and specificity of both case definitions was lower in children aged 0–15 years than in older age groups, particularly for the current case definition. Sensitivity of the current case definitions remained stable (81-85%) between June 2020 through June 2021 apart from a decrease in February to April 2021 (66%, 95% CI 55-76%). In contrast the sensitivity of the proposed case definition remained stable over the entire period (87-95%). Specificity of both case definitions generally increased over time although the current case definition dipped in May-June 2021. The periods of lowest specificity coincide with periods when other acute respiratory viruses were more common in the population, such as during a large national outbreak of Rhinovirus in children in late summer and autumn 2020 ^12^. The PPV (the proportion of those meeting the clinical case definition who test positive) was 15% for the current UK case definition and 12% for the broader definition. PPV estimates were substantially lower (between 5-6%) in the youngest age group and during June through September 2020 and in February through June 2021, corresponding with the age group and time periods with the lowest disease rates. NPV (the proportion of those with illnesses not meeting the clinical case definition who tested negative) was 96% for the current UK case definition and 97% for the broader case definition. The number of illnesses meeting the broader case definition was 1.7-fold higher than those meeting the current definition.

## Discussion

We characterised the symptom profiles and estimated the accuracy of clinical case definitions for COVID-19 among community cases arising in a large, prospective, population-based cohort study based in the UK. All symptoms asked about were more frequently reported, and when present were generally more severe and longer-lasting, in COVID-19 illnesses compared to non-COVID-19 illnesses. Individually, cough and some constitutional symptoms including headache, muscle ache and fatigue presented early in the course of illness and had moderate sensitivity and specificity as they were common in both test positive and test negative illnesses. In contrast, fever and loss of or altered sense of smell or taste had a lower sensitivity but higher specificity as they were not as common in COVID-19 illnesses but were even less common in non-COVID-19 illnesses and loss of or altered sense of smell or taste also presented slightly later in the course of illness. The combination of symptoms in the current UK case definition had a higher sensitivity (81%) than any individual symptom, with a specificity of 47%. Adding additional symptoms to the case definition could lead to earlier case identification and higher sensitivity, but at the cost of specificity and consequently, a substantial increase in the number of non-COVID-19 illnesses eligible for testing. For example, when we compare the broader case definition to the current UK case definition, cases on average met the case definition 0.3 days earlier and there was a moderate increase in sensitivity to 93% but at a much lower specificity of 27%, with 1.7 times more illnesses eligible for testing. 

Strengths of this work include the prospective daily recording of a wide range of symptoms across a large community cohort and linkage of these to self-reported swab results ascertained on a weekly basis. This should maximise the accuracy of symptom data amongst swabbed participants. While not fully representative of the population of England and Wales, our sample includes participants in every local authority area; however, there is a moderate overrepresentation of those aged over 65 and an underrepresentation of those in more deprived areas ^13^. Whilst we collected information on a very wide range of symptoms, testing primarily relied on that conducted through the national TTI programme meaning that those meeting the current case definition are more likely to be tested. This is likely to lead to an overestimation of the sensitivity of the current UK case definition. This bias towards testing illnesses meeting the case definition however was reduced over time. This change in testing behaviour and the drop in sensitivity seen between February-April 2021 may be attributed, at least in part, to the roll-out and widespread availability and use of at-home lateral flow testing to the wider population from March 2021. The availability and ease of at-home lateral flow devices may have lowered individuals’ threshold for testing, leading to more mild illnesses being tested. This phenomenon would not explain the subsequent apparent rise in sensitivity from May-June 2021, although the confidence intervals for the sensitivity estimates between the February-April and May-June strata overlap (55%-76% and 65%-94% respectively) so the increased sensitivity could be due to chance. Due to limited data during the relevant time period, we are unable to assess whether any of the apparent changes in the sensitivity or specificity in May-June 2021 may be due to differing symptom profiles between the Alpha Variant of Concern (which was the dominant UK strain in winter and early spring 2021) and the Delta Variant of Concern (which became the dominant variant during May 2021) ^14^. 

COVID-19 is difficult to distinguish from other respiratory infections or common ailments on the basis of symptoms alone. Also, a high proportion of infections are asymptomatic or have a pre-symptomatic phase when transmission can occur ^15, 16^. As such, systems to identify symptomatic cases, test them, and isolate cases and their contacts can only ever reduce, rather than prevent all transmission. These programmes, when part of a broader programme of non-pharmaceutical interventions, can however contribute to control of infection and may be particularly effective when introduced during periods of lower incidence. For example, countries that combined early and strict border controls, intensive testing, and rapid triggering of lockdowns have had substantially lower COVID-19 transmission and mortality than other countries such as the UK ^17^.

The success of testing and isolation programmes is dependent on public understanding and engagement. Data from behavioural surveys in England show only 51.5% of participants knew the symptoms that testing is recommended for, only 18.0% sought testing if they had the symptoms and only 42.5% fully adhered to self-isolation ^18^. Engagement was lower in younger people and amongst those in financial hardship ^18^. Engagement with population level asymptomatic testing using lateral flow testing has also been shown to be low, particularly in socioeconomically disadvantaged areas ^19^.

Policy makers considering which symptoms might prompt testing, tracing and isolation need to balance the availability of testing capacity at different stages of the pandemic, the speed with which samples can be taken and results returned, the harms incurred by asking large numbers of people who do not have COVID-19 and their contacts to self-isolate whilst awaiting test results, the consistency and simplicity of public health messaging and the likely public engagement with the system. Alteration of symptom profiles triggering testing and isolation may have less impact than other approaches to increase uptake and engagement, and to ensure timely and effective tracing and isolation of household and non-household contacts, including through providing financial and non-financial support for people to isolate ^20^.

The fact that COVID-19 may present as any of a very wide range of symptoms, or with no symptoms at all, is one of the key challenges in implementing successful TTI systems. Low levels of engagement also limit effectiveness. This emphasises the importance of not placing undue reliance on such systems as a mechanism to allow relaxation of other social distancing measures and the critical importance of protecting populations globally through immunisation. 

## Data availability

### Underlying data

We aim to share aggregate data from this project on our website and via a "Findings so far" section on our website - https://ucl-virus-watch.net/. We are sharing individual record level data on the Office of National Statistics Secure Research Service, and given the sensitive content in our dataset for this study, we cannot release the data at the individual level. In sharing the data we will work within the principles set out in the UKRI Guidance on best practice in the management of research data. Access to use of the data whilst research is being conducted will be managed by the Chief Investigators (ACH and RWA) in accordance with the principles set out in the UKRI guidance on best practice in the management of research data. Data access requests to data can also be made directly to the Virus Watch chief investigators (ACH or RWA) at the following email address: viruswatch@ucl.ac.uk.

### Extended data

Zenodo: UCL-Public-Health-Data-Science/Symptom-profiles-and-accuracy-of-clinical-case-definitions-for-COVID-19-: first release, https://doi.org/10.5281/zenodo.5888582 ^11^.

This project contains the following extended data:

- Fragaszy_et_al_Symptom Profiles_Appendix_for_subm.docx (document containing the following information and supplementary figures and tables:
  ○ Additional methods
  ○ Figure S1. Two by two contingency table and equations used to calculate test characteristics of the clinical case definitions compared to swab test results (gold standard)
  ○ Figure S2. Percent of illnesses with a swab test result by month, overall and by UK COVID-19 Case Definition status
  ○ Figure S3. Ratio of swabbing among illnesses meeting the UK COVID-19 case definition to those not meeting the case definition over time
  ○ Figure S4. Distribution of illness duration* by age group among confirmed COVID-19 illnesses
  ○ Table S1. Details of symptoms and symptom groupings
  ○ Table S2. Speed of symptom onset, proportion of all community cases tested, and the test characteristics of each symptom
  ○ Table S3. Severity of symptoms among swab-confirmed COVID-19 and non-COVID-19 (swab-negative) illnesses
  ○ Table S4. Duration of illness among swab-confirmed COVID-19 illnesses and non-COVID-19 (swab-negative) illnesses, overall and stratified by age group and sex)
- symp-stat01_Table_1_v10.do
- symp-stat02_v5_Figure_1.do

Data are available under the terms of the Creative Commons Attribution 4.0 International license (CC-BY 4.0).
