# Supplementary material for: Symptom profiles and accuracy of clinical case definitions for COVID-19 in a community cohort: results from the Virus Watch study
Source: Wellcome Open Res. 2022 Mar 10;7:84. [Version 1] doi: 10.12688/wellcomeopenres.17479.1 (PMC10514573; doi:10.12688/wellcomeopenres.17479.1)
Supplement: Peer review history

# Reviewer report: Reviewer response for version 1

**DOI:** 10.21956/wellcomeopenres.19328.r66603

**Published:** 2023-09-21

**Authors:** Michael E DeWitt

**Affiliations:** 1 Department of Internal Medicine, Section on Infectious Diseases, Wake Forest University School of Medicine, Winston-Salem, North Carolina, USA

Fragaszy and colleagues describe symptomology of SARS-CoV-2 infections in a prospectively enrolled community cohort between June 2020 and June 2021. This research provides important information on the role of symptom-based surveillance and guidance. I have several minor comments.

 Discussion:

 It is likely worth discussing or at least mentioning the mixture of testing modalities and the bias that they could introduce (e.g., sensitivity/specificity of lateral flow assays vs PCR). I'm thinking more generally between the test but also regarding the likelihood of detection given the timing of the test (see the Hellewell citation ^1^). 

 One point of clarification that the mean onset of symptoms refer to non-index symptoms (i.e., at least one new symptom trigger a symptomatic event and the subsequent symptom onsets, like cough at day 2 as in table S2) were after the index symptom?

 Could you include a reference to the definition mild/moderate/severe as used in the supplemental tables? I imagine it was in the diary, but I could not find the definition (and theoretically severe symptoms would end up in hospital and would have some reporting bias).

 Fantastic work!

Is the work clearly and accurately presented and does it cite the current literature?

Yes

If applicable, is the statistical analysis and its interpretation appropriate?

Yes

Are all the source data underlying the results available to ensure full reproducibility?

Yes

Is the study design appropriate and is the work technically sound?

Yes

Are the conclusions drawn adequately supported by the results?

Yes

Are sufficient details of methods and analysis provided to allow replication by others?

Yes

Reviewer Expertise:

Epidemiology

I confirm that I have read this submission and believe that I have an appropriate level of expertise to confirm that it is of an acceptable scientific standard.

## References

[1] : Estimating the effectiveness of routine asymptomatic PCR testing at different frequencies for the detection of SARS-CoV-2 infections. *BMC Med* .2021;19(1) : 10.1186/s12916-021-01982-x 106 10.1186/s12916-021-01982-x 33902581PMC8075718

# Reviewer report: Reviewer response for version 1

**DOI:** 10.21956/wellcomeopenres.19328.r56355

**Published:** 2023-04-28

**Authors:** Benjamin J. Cowling

**Affiliations:** 1 WHO Collaborating Centre for Infectious Disease Epidemiology and Control, School of Public Health, The University of Hong Kong, Hong Kong, Hong Kong

Fragaszy and colleagues describe the symptom profiles of COVID-19 based on data from a large community cohort study. This is valuable work. I had mainly minor comments.

**Major comment:**

1. To what extent has clinical symptomatology changed over time, either with changes in the variants, or with immunity following vaccination doses and/or infections? There are many media reports of "symptom X" is more common with "variant Y". For example in April 2023 "red eye" is being mentioned as a symptom appearing with the latest Omicron subvariant (I am not saying this observation is true, just that it is being reported in the media). This issue of potentially changing symptom profiles should perhaps be mentioned in various places, starting in the Introduction.

**Minor comments:**

1. Abstract background - "...Test, Trace and Isolate (TTI)..." is TTI still being done? Wouldn't antiviral treatment largely be based on testing, either by LFT or PCR, given that testing is now widely available?
2. Abstract results and main text Results, please clarify the variants involved, or at least the predominant variants during various periods, for example when was Alpha or Delta predominant?
3. Do you have data on the subset of illnesses which led to medical attention, and how was the symptom profile in that subset of cases?

Is the work clearly and accurately presented and does it cite the current literature?

Yes

If applicable, is the statistical analysis and its interpretation appropriate?

Yes

Are all the source data underlying the results available to ensure full reproducibility?

Yes

Is the study design appropriate and is the work technically sound?

Yes

Are the conclusions drawn adequately supported by the results?

Yes

Are sufficient details of methods and analysis provided to allow replication by others?

Yes

Reviewer Expertise:

Epidemiology

I confirm that I have read this submission and believe that I have an appropriate level of expertise to confirm that it is of an acceptable scientific standard.
